# Editorial: The impact of robotic technologies on customer experience and adoption

**DOI:** 10.3389/frobt.2026.1857216

**Published:** 2026-05-11

**Authors:** Anshu Saxena Arora, Amit Arora, John McIntyre

**Affiliations:** 1 School of Business and Public Administration, University of the District of Columbia, Washington, DC, United States; 2 Scheller College of Business, Georgia Institute of Technology, Atlanta, GA, United States

**Keywords:** expressive interaction, fear typologies, multimodal representation, robot experience, robot-scripted service workflows, socio-behavioral interpretability

This Research Topic, Frontiers | *The impact of robotic technologies on customer experience and adoption*, brings together five contributions that collectively explain how users’ psychological responses, situational context, and specific interaction design decisions jointly shape customer experience and adoption outcomes in service and social robotics. Robots are increasingly present in customer-facing contexts, from healthcare and hospitality to retail and public services, and recent work underscores that making AI-powered service robots more transparent can improve customers’ situational awareness but also heighten privacy worries and, when safeguards seem inadequate, reduce their willingness to engage with such systems (Hu and Min, 2025; Muller Dardelin et al., 2026). Users must feel safe, respected, and supported; they must understand what a robot is doing and why; and they must experience interactions that fit the surrounding social context, including expectations about how visible and “legible” robot decision-making should be in shared public spaces, where design transparency has been shown to shape trust and customers’ willingness to share data with robots (Aryania et al., 2026). Building on these broader findings, we are providing some research themes that erupted from the collection of articles in the Research Topic with future research questions tied to each contribution.

Socio-Behavioral Interpretability as a Driver of Customer Experience. Building on the opening article of the Research Topic, Frontiers | *Transforming customer experience in social robotics through explainable and interpretable artificial intelligence over a decade*, the first research theme emerging from a decade-long synthesis of Explainable AI (XAI) and Interpretable AI (IAI) in social robotics focuses on socio-behavioral interpretability as a key driver of customer experience Arora et al. Over time, XAI and IAI in social robots have shifted from purely technical self-explanations toward socio-behavioral interpretability that aligns explanations with users’ goals, emotions, and social norms, supporting trust, emotional comfort, and long-term user experience/customer experience (UX/CX). Future research should examine how different explanation styles (e.g., needs-based, causal, contrastive, or counterfactual) differentially affect immediate satisfaction versus long-term trust, loyalty, and relationship depth across domains such as healthcare, retail, and education. A related question is how to calibrate socio-behavioral interpretability so robots are transparent enough to foster trust while avoiding over-trust or unhealthy attachment, especially among vulnerable users such as older adults or children. The paper’s four eras show a trajectory from rule-based transparency to integrated UX/CX and ethical architectures embedded in real service ecosystems. This raises questions about how CX outcomes and adoption patterns differ when “foundational” versus “advanced” XAI/IAI robots operate in the same context, and what governance and design guidelines managers need to select, upgrade, and retire robots over time while maintaining a coherent, trustworthy brand experience.

Fear Typologies as Regulators of Technology Acceptance. The second research theme emerges from the second article of this Research Topic, Frontiers | *Exploring fear in human-robot interaction: a scoping review of older adults experiences with social robots* that focuses on multidimensional fear typologies as hidden regulators of technology acceptance Elsheikh et al. The review shows that older adults’ fears are not a single construct but span privacy and autonomy concerns, trust and reliability issues, emotional and ethical discomfort, usability challenges, fear of dependence, unfamiliarity with technology, and Uncanny Valley reactions, each influenced by robot design, prior technology experience, cultural background, and care context. Future research should investigate how specific constellations of these fear types predict distinct adoption trajectories, such as outright rejection, reluctant use, or eventual normalization of social robots across home, community, and institutional eldercare settings. A closely related question is how to design robust, standardized, multi-method measures of fear, combining self-report scales, behavioral observation, and physiological indicators that can be used to monitor fear dynamics longitudinally during real-world deployments and to evaluate the impact of design or implementation changes on older adults’ emotional safety and engagement with robots.

Multimodal Representations as Catalysts of Social Connection and Acceptance. A third research theme focused on social connection and acceptance, emerges from this article of the Research Topic, *Frontiers | How multimodal narrative and visual representations of human-like service robots shape attitudes and social connection*, which treats media portrayals as levers for shaping customers’ initial stance toward robots Daruwala et al. The study shows that exposure to combined visual and narrative interventions (e.g., imagery, short videos of human-like gestures, and vignettes of everyday service encounters) significantly reduces negative attitudes, lowers negative affect, and increases perceived closeness to service robots, highlighting the power of empathetic, humanizing portrayals to foster openness before any direct interaction occurs. Future research should investigate how specific multimodal configurations (high versus moderate anthropomorphism, task-focused versus relationship-focused narratives, or varying levels of expressed empathy) differentially shape trust, perceived warmth, and willingness to interact across sectors such as caregiving, hospitality, and retail. A related question is how stable and transferable these media-induced attitude shifts are over time and across contexts: do early positive representations create durable expectations that enhance real-world human-robot interaction, or do they risk generating mismatched expectations and disappointment when actual robots fall short of their mediated portrayals?

Social Context and Expressive Interaction as Co-Determinants of Public Service Robot Experience. A fourth research theme emerges from *Frontiers | How the presence of others shapes the user experience of service robots*, which reconceptualizes public human-robot interaction as social performances shaped by observers and psychological needs rather than dyadic interactions alone Tretter et al. The study shows that when people imagine interacting with a service robot in front of close others, relatedness needs become salient and drive preferences for more expressive, visible interactions, whereas in the presence of strangers, popularity needs are foregrounded and lead to a desire for expressivity mainly when success can be claimed as one’s own achievement. Future research should examine how real-time variations in social configuration (alone, with friends or family, or surrounded by strangers) and perceived performance risk jointly shape preferred interaction modalities (e.g., voice versus touch), expressivity levels, and willingness to engage with service robots in actual public settings such as cafés, airports, and hospitals. A related question is how designers and service providers can operationalize these insights into adaptive interaction policies, such as robots that sense social context and dynamically modulate expressivity while avoiding embarrassment, social exclusion, or unwanted visibility for users who prefer more discreet interactions.

Robot Experience and Scripted Service Workflows in Public Health. A fifth research theme arises from Frontiers | *Effectiveness of conversational script optimization by intelligent consultation robots on daily work efficiency in vaccination clinics*, which foregrounds robot experience as scripted service workflow and operational CX Zhou et al. Rather than emphasizing embodiment or appearance, this study explores how iterative optimization of an intelligent consultation robot’s dialogue guidance strategy, moving from open-ended prompts to concise, structured two-round scripts, directly reshapes both back-stage performance (automation rates, human-transfer rates, and the number of routine issues resolved) and front-stage experience, including shorter wait times and improved satisfaction. Future research should examine how different script architectures (for example, tightly guided versus flexible flows, early versus late escalation to human staff, and proactive versus user-initiated handoff) influence patients’ perceived clarity, reassurance, and trust, alongside staff workload and throughput, in vaccination clinics and other high-volume, information-intensive services. A related question is how to conceptualize and measure “robot experience” from the customer’s perspective, integrating perceived responsiveness, conversational smoothness, error handling, and handoff quality, and how these robot-experience metrics interact with broader CX outcomes such as confidence in public health services, willingness to reuse automated channels, and equity of access for users with different levels of health literacy or digital familiarity.

Together, these contributions suggest a practical agenda for advancing robot-enabled CX and adoption. First, explainability and interpretability must be treated as UX and CX capabilities, not only technical properties, tailored to domain expectations and ethical constraints (Aryania et al., 2026). Second, emotional barriers such as fear and discomfort require proactive design and implementation strategies, including transparency, supportive interaction, and participatory development, particularly for older adults and other vulnerable users (Li et al., 2025). Third, acceptance is shaped by narrative and imagery as well as direct experience, creating opportunities and responsibilities for accurate, empathetic communication about anthropomorphic service robots. Fourth, social context matters, especially in public settings where observers can shift user motivations and preferred interaction styles by activating needs for relatedness or popularity. Finally, adoption in real services depends on interaction details such as robot’s dialogue structure and escalation rules, where iterative conversational optimization can produce measurable improvements in efficiency and perceived experience (Muller Dardelin et al., 2026).

We hope this Research Topic stimulates further interdisciplinary work that connects robotics, human-robot interaction, marketing, psychology, and service operations. Future research would benefit from longitudinal field studies that link design choices to sustained trust and continued use, cross-cultural comparisons that clarify boundary conditions for fear and acceptance, and standardized measurement approaches that connect operational metrics to customer experience outcomes (Aryania et al., 2026; Muller Dardelin et al., 2026).
